# 

**Published:** 1902-06

**Authors:** 


					﻿EX
o_
LU
OC
o_
LU
CD
*C
GO
O_
•
1
o
<
u
o
ID
CM
6
2
o"
o
o>
o7
0)
■
00
0)
I
N
0)
i
(0
0)
CD
LL-
O
GO
LU
OU
CD
CD
=3
ca
LEADS VETERINARY JOURNALISM IN AMERICA.
Vol* XXIII.
JUNE, 1902.
No. 6
PUBLISHED MONTHLY AT $3 A YEAR. SINGLE COPIES, 30 CENTS.
FOREIGN SUBSCRIPTIONS $3.50 PER YEAR.
THE JOURNAL
OF
Comparative Medicine
AND
VETERINARY ARCHIVES
EDITED AND PUBLISHED BY
W. HORACE HOSKINS, D.V.S.
DO
O
c
z
□
o
o
2
m
CD
o
"n
co
o
©
m
<
-n
O
©
□
m
r
<
m
©
ALL COMMUNICATIONS AND PAYMENTS SHOULD BE ADDRESSED TO
Dr. W. Horace Hoskins, Managing editor, 345a Ludlow St., Philadelphia, Pa.
Copies may be had on order of all news-dealers at home and abroad.
AN ADVERTISING MEDIUM UNEQUALLED IN THE VETERINARY FIELD
				

